# Supplementary material for: Polyketide synthase gene clusters in the ericoid mycorrhizal fungus Oidiodendron maius: genome-wide prediction, expression profiling, and functional characterization of knockout mutants
Source: Front Plant Sci. 2026 Sep 16;17:1924430. doi: 10.3389/fpls.2026.1924430 (PMC13623903; doi:10.3389/fpls.2026.1924430)
Supplement: Supplementary file 1 [file SupplementaryFile1.zip › Supplementary Materials/Supplementary Folder 1.zip!/Oidma1_AssemblyScaffolds_Repeatmasked/knownclusterblast/cluster39/jgi.p_Oidma1_59248_mibig_hits.html]

| MIBiG Protein | Description | MIBiG Cluster | MiBiG Product | % ID | % Coverage | BLAST Score | E-value |
| --- | --- | --- | --- | --- | --- | --- | --- |
| ACH72896.1 | AflO | BGC0000011 | Aflatoxin/sterigmatocystin\_biosynthetic\_gene\_cluster | 33 | 100.0 | 235 | 1.4e-61 |
| AAS90034.1 | OmtB | BGC0000008 | Aflatoxin\_biosynthetic\_gene\_cluster | 33 | 97.3958333333 | 225 | 1.1e-58 |
| AAS90103.1 | OmtB | BGC0000006 | Aflatoxin\_biosynthetic\_gene\_cluster | 33 | 97.3958333333 | 225 | 1.4e-58 |
| AAS90012.1 | OmtB | BGC0000007 | Aflatoxin\_biosynthetic\_gene\_cluster | 33 | 97.3958333333 | 224 | 1.8e-58 |
| BAE71328.1 | o-methyltransferase\_B | BGC0000004 | Aflatoxin\_biosynthetic\_gene\_cluster | 33 | 97.3958333333 | 224 | 1.8e-58 |
| AAS90080.1 | OmtB | BGC0000010 | Aflatoxin\_biosynthetic\_gene\_cluster | 33 | 97.3958333333 | 223 | 5.3e-58 |
| AAS90060.1 | OmtB | BGC0000009 | Aflatoxin\_biosynthetic\_gene\_cluster | 32 | 97.1354166667 | 217 | 2.2e-56 |
| ctg1\_orf003 |  | BGC0000685 | Brassicicene\_C\_biosynthetic\_gene\_cluster | 33 | 76.5625 | 198 | 1.8e-50 |
| ACZ66252.1 | APS6 | BGC0000304 | Apicidin\_biosynthetic\_gene\_cluster | 31 | 92.96875 | 168 | 1.2e-41 |
| AKD43502.1 | O-Methyltransferase | BGC0001409 | Dutomycin\_biosynthetic\_gene\_cluster | 26 | 91.1458333333 | 91 | 1.9e-18 |
| CCE67071.1 | methyltransferase | BGC0001242 | Fusarubin\_biosynthetic\_gene\_cluster | 29 | 52.8645833333 | 88 | 2e-17 |
| ACN64847.1 | PokMT3 | BGC0001061 | Polyketomycin\_biosynthetic\_gene\_cluster | 26 | 89.3229166667 | 84 | 2.3e-16 |
| AFD30960.1 | CrmM | BGC0000966 | Caerulomycin\_A\_biosynthetic\_gene\_cluster | 22 | 93.2291666667 | 80 | 4.3e-15 |
| AAM70356.1 | CalO6 | BGC0000033 | Calicheamicin\_biosynthetic\_gene\_cluster | 27 | 53.125 | 75 | 1.8e-13 |
| AHI58817.1 | WelM | BGC0000668 | Welwitindolinone\_biosynthetic\_gene\_cluster | 21 | 88.28125 | 61 | 2.7e-09 |
| AAD32742.2 | MmcR | BGC0000915 | Mitomycin\_biosynthetic\_gene\_cluster | 29 | 38.5416666667 | 60 | 6e-09 |
| sln4 | methyltransferase | BGC0001230 | Salinamides\_biosynthetic\_gene\_cluster | 28 | 42.96875 | 56 | 8.6e-08 |
